# Rowing Crew Coordination Dynamics at Increasing Stroke Rates

**DOI:** 10.1371/journal.pone.0133527

**Published:** 2015-07-17

**Authors:** Laura S. Cuijpers, Frank T. J. M. Zaal, Harjo J. de Poel

**Affiliations:** Center for Human Movement Sciences, University Medical Center Groningen, Groningen, University of Groningen, the Netherlands; Purdue University, UNITED STATES

## Abstract

In rowing, perfect synchronisation is important for optimal performance of a crew. Remarkably, a recent study on ergometers demonstrated that antiphase crew coordination might be mechanically more efficient by reducing the power lost to within-cycle velocity fluctuations of the boat. However, coupled oscillator dynamics predict the stability of the coordination to decrease with increasing stroke rate, which in case of antiphase may eventually yield breakdowns to in-phase. Therefore, this study examined the effects of increasing stroke rate on in- and antiphase crew coordination in rowing dyads. Eleven experienced dyads rowed on two mechanically coupled ergometers on slides, which allowed the ergometer system to move back and forth as one ‘boat’. The dyads performed a ramp trial in both in- and antiphase pattern, in which stroke rates gradually increased from 30 strokes per minute (*spm*) to as fast as possible in steps of 2 *spm*. Kinematics of rowers, handles and ergometers were captured. Two dyads showed a breakdown of antiphase into in-phase coordination at the first stroke rate of the ramp trial. The other nine dyads reached between 34–42 *spm* in antiphase but achieved higher rates in in-phase. As expected, the coordinative accuracy in antiphase was worse than in in-phase crew coordination, while, somewhat surprisingly, the coordinative variability did not differ between the patterns. Whereas crew coordination did not substantially deteriorate with increasing stroke rate, stroke rate did affect the velocity fluctuations of the ergometers: fluctuations were clearly larger in the in-phase pattern than in the antiphase pattern, and this difference significantly increased with stroke rate. Together, these results suggest that although antiphase rowing is less stable (i.e., less resistant to perturbation), potential on-water benefits of antiphase over in-phase rowing may actually *in*crease with stroke rate.

## Introduction

Crew rowing is often quoted as the prime example of real-life joint action, interpersonal coordination dynamics and synchronisation (e.g., [1–5]), and for group processes in general as well (e.g., [6, 7]). The degree of synchronisation between the rowers in a boat is generally regarded as an important determinant for optimal crew performance (e.g., [8, 9]). Even a team of individually strong and technically skilled rowers will probably not win the race if they do not properly coordinate their movements together. Researchers, coaches and rowers agree that to achieve optimal performance of the crew, rowers must row in perfect synchrony [10]. Despite the well-known tendency of humans to synchronize their movements (e.g., [11]), though, rowing in sync does not come naturally. Inexperienced crews often have difficulties row in a common rhythm, and it often takes many years to perfect the synchrony of the crew. In the current study we examine such crew synchronisation processes. Before testing on water, we chose to first study the coordination of rowing dyads in a controlled laboratory setting. More specifically, using a setup of coupled rowing ergometers (see below), the current study investigated the effect of movement frequency on crew coordination.

With regard to crew coordination it is important to note that a stroke cycle consists of two phases, namely the *drive*, during which force is applied to the blade in order to move the boat relative to the water, and the *recover*, during which the rowers return to their former position. Hence, there is discontinuous propulsion. Moreover, the rowers are seated on sliding seats, which allows for the essential contribution of the strong leg extensor muscles to the propulsion. In each stroke cycle the relatively heavy crew concurrently pushes off against the foot stretchers, thereby causing the relatively light boat to accelerate in the opposite direction of the crew. As a result, the velocity of the boat fluctuates within each rowing cycle, which implies a 5–6% power loss [12, 13, 14].

Interestingly, theoretically, an out-of-phase crew coordination pattern may minimise this power lost to velocity fluctuations of the boat [15]. By perfectly alternating their strokes, the produced forces and associated movements of the rowers perfectly counteract each other, yielding the net centre of mass of the crew to stay close to the centre of mass of the boat. As a result, the shell velocity remains near to constant over the whole rowing cycle. A recent off-water study in which dyads rowed on mechanically linked ergometers at a rate of 36 strokes per minute (*spm*) confirmed that, compared to in-phase rowing, antiphase crew coordination drastically decreased ergometer displacement, and demonstrated that the 5–6% power lost to velocity fluctuations was indeed regained in the antiphase rowing condition [13].

Although antiphase rowing might be beneficial due to reduction of velocity fluctuations of the boat, the question is how stable antiphase patterns can be performed at the higher stroke rates as typically seen in racing. From coordination dynamics literature [16–20] it is known that an increase in movement rate may destabilise the coordinative performance, especially for antiphase coordination [17–19], which at a critical frequency yields transitions towards in-phase coordination [16–18]. As stroke rates during a race can reach values over 40 *spm*, it is interesting to examine whether antiphase crew synchronisation can be maintained with sufficient stability at the highest movement frequencies. For these reasons, the present study set out to systematically examine both in-phase and antiphase crew rowing at increasing stroke rates, from a coordination dynamics perspective.

### Coordination dynamics

The coordination between people has been studied for a range of cyclic movement tasks, such as moving hand-held pendulums [21–22], rocking chairs [23], joint stepping [24] and side-by-side walking [25] (for an overview, see e.g., [26]). Given the cyclical nature of the rowing act, along with the fact that it has to be performed in coordination with other rowers, crew rowing movements can be modelled as coupled oscillations. As such, coordination dynamics offers a pertinent theoretical approach to study crew synchronisation. From this perspective, the coordination between two oscillatory components can be described in terms of the relative phase (*ϕ*), formulated as:

$$\phi =\theta_{1}-\theta_{2}$$ (1)

with *θ*_1_ and *θ*_2_ depicting the phase angle of each oscillator (i.e. were it resides in its cycle from 0° to 360°). From studies on bimanual interlimb coordination, it is known that people are generally able to perform two stable coordinative patterns, namely in-phase coordination (*ϕ* = 0°), wherein the rhythmic hand movements perfectly coincide, and antiphase coordination (*ϕ* = 180°), in which the rhythmic movements perfectly alternate with half-a-cycle difference. Other coordinative patterns are unstable without training [27].

For both within- [16–17] as between-person coordination [4, 18–20] it has been shown that when people start coordinating in an antiphase pattern, an increase in movement frequency typically results in a transition into in-phase coordination. Importantly, when people start in in-phase, no transition into another pattern (e.g., antiphase) occurs. The fact that this phenomenon is observed in both within- and between-person coordination indicates that the source of coupling (neural, perceptual, or otherwise) is immaterial for the synchronisation process [28, 29].

As an account for these behavioural phenomena, Haken, Kelso and Bunz [17] formulated a model of two non-linearly coupled limit cycle oscillators from which an equation of motion could be derived that captured the rate of change in relative phase angle between the two oscillating components, following:

$$\dot{\phi }=-a\;\text{sin}\;\phi -2b\;\text{sin}\;2\phi$$ (2)

with *a* affecting the attractor strength of in-phase coordination, and *b* affecting the attractor strength of both in- and antiphase coordination. The ratio *b/a* is directly related to the movement frequency. Given Eq 2, at low frequencies (i.e., *b/a* > .25) the model has two stable attractors, namely in-phase and antiphase coordination, while at higher movement frequencies (i.e., *b/a* < .25) the attractor strength of both in- and antiphase decreases with increasing frequency, although this decrease is stronger for antiphase than for in-phase coordination. This frequency effect for in- and antiphase has been supported in numerous bimanual studies (see [30] for an overview) and between-persons studies [18–19]. The coupled oscillator model shows that, due to coupling forces, at a certain critical frequency the antiphase pattern is not stable anymore and the coordination is compelled towards a transition to the remaining stable in-phase pattern. Would this also show up in crew rowing at increasing stroke rates?

### In-phase vs. antiphase crew coordination

De Brouwer et al. [13] showed that, consistent with previous studies on coordination dynamics, antiphase crew coordination (as measured by the phase relation between the rowers’ centres of mass) was less accurate and less consistent compared to conventional in-phase crew coordination. Still, although the dyads in that study performed an antiphase pattern for the first time ever, they demonstrated strikingly little difficulty rowing in antiphase, even at the rather high stroke rate of 36 *spm*. This clearly confirmed the intrinsic stability of the interpersonal antiphase pattern and the ease to perform this pattern. However, in their steady state trials, one dyad showed a brief breakdown of the antiphase pattern towards in-phase crew coordination [13]. This suggests that despite the apparent ease with which antiphase rowing was performed, this pattern is still less stable and therefore more prone to perturbations of any kind, which may break down the coordination pattern.

**Fig 1:**
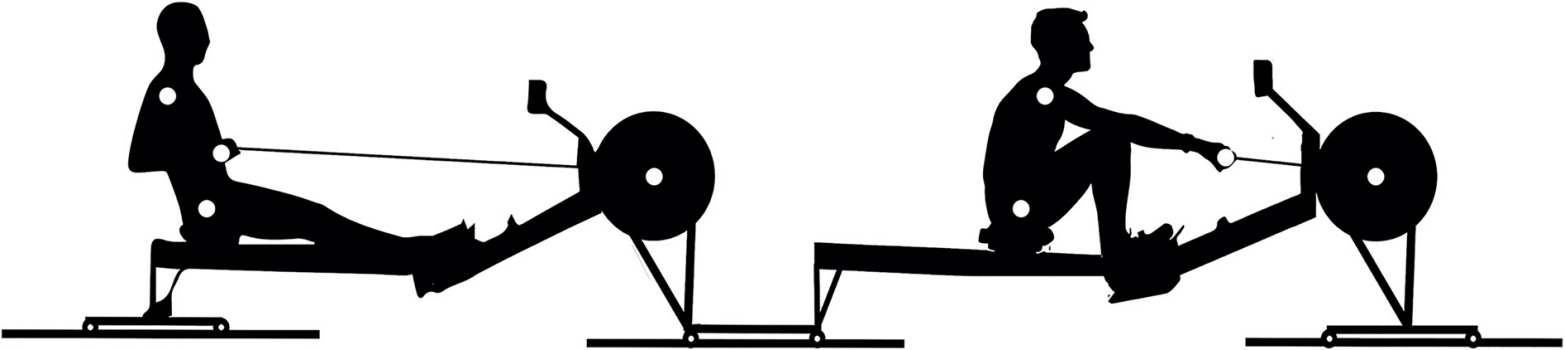
Experimental Setup. Note that only the markers placed on the right side are shown. See also S1 Movie and S2 Movie for exemplary movies.

For obvious reasons, most studies regarding interpersonal coordination emphasise perceptual coupling (mainly visual) in mediating synchronisation processes [11, 29–32]. In crew rowing, though, there is also a direct *physical* coupling between the rowers, namely through the boat that they share. To date not many studies on interpersonal coordination have addressed this type of coupling. Crew rowing may therefore serve as an interesting experimental task to provide insights for coordinative behaviour of mechanically coupled individuals (see also [33]). To create the physical coupling and address the effect of crew coordination on ‘boat’ movements, similar to De Brouwer et al. [13], we used two rowing ergometers serially placed on slides to allow these to move back and forth as one ‘boat’ (see Fig 1 and *Experimental Setup*). (Note that with ‘boat’ (between apostrophes) we refer to the mechanically linked ergometers.) Obviously, rowing on water differs from rowing on linked ergometers (see *Discussion*). Yet, the kinematics of ergometer rowing are largely similar to those of on-water rowing [34], particularly for free-floating ergometers as used in the present study [35–36]. In sum, given the above, we expect faster stroke rates to involve poorer crew synchronisation for both in- and antiphase rowing, with a stronger effect for antiphase. Most importantly, antiphase crew coordination is expected to break down with increasing stroke rate.

## Method

### Participants

Eleven male rowing dyads participated in the experiment (age 24 ± 5 years; length 1.86 ± 0.05 m; mass 76.86 ± 8.27 kg; rowing experience at club level 5 ± 3 years). Nine dyads signed up for the experiment as actual teammates, while the other two dyads were composed based on availability for the experiment and matched for body mass. All participants provided written informed consent prior to participation. The individuals in the movies in the supplementary material (S1 Movie and S2 Movie) have given additional written informed consent (as outlined in the PLOS consent form) to publish video material of their experimental trials. The Ethics Committee of the Center for Human Movement Sciences, University Medical Center Groningen approved the study that was conducted according to the principles expressed in the Declaration of Helsinki.

### Experimental setup

Trials were performed using two mechanically coupled rowing ergometers (Type D, Concept2, USA) serially placed on slides (see Fig 1). This setup allows the ergometers to move with respect to the floor as a single ‘boat’, reminiscent to on water rowing. The resistance of the ergometer flywheels was set at a level that reflected on-water blade resistance. Dyads determined and set the resistance at an aerobic constant between 0.96∙10^−4^ kg∙m^2^ and 1.20∙10^−4^ kg∙m^2^ (i.e., drag factor 96–120). Rowers within a dyad rowed at the same drag factor.

The kinematics of the rowers and ergometer system were recorded with a Vicon Motion Analysis System (Vicon Motion Systems, Inc., Centennial, CO), using 17 retro-reflective markers. Three markers were placed on the ergometer of the stroke rower, the remaining markers were placed on both sides- and on the middle of each handle and on both left and right shoulder (acromion) and hip (greater trochanter) of each rower, as depicted in Fig 1. Using 8 infrared cameras placed around the measurement volume, the markers’ 3D-trajectories were collected at a sample rate of 200 Hz.

### Protocol

To warm up and get familiar with the experimental set up, each dyad started with rowing five minutes in in-phase coordination and five minutes in antiphase coordination at a self-chosen stroke rate (about 20–24 *spm*), including about 30 *s* of rowing at a higher stroke rate (>30 *spm*) for each condition. After a short break, each dyad performed a two-minute in-phase and a two-minute antiphase trial at 30 *spm* in counterbalanced order with at least five minutes of rest in between. Next, for both in- and antiphase crew coordination the dyads performed a ramp trial in which they were instructed to increase stroke rate every 30 *s* with 2 *spm*, starting from 30 *spm* until the dyad could not increase the stroke rate any further. The rowers received feedback about stroke rate on a monitor (PM4, Concept 2, USA; see Fig 1). Exemplary movies of in- and antiphase rowing are provided in S1 and S2 Movies.

### Data analysis

Since in the current study we were specifically interested in the occurrence of coordination breakdowns and the stability of crew coordination over increasing stroke rates, we only report the analysis of the ramp trials. Movement data were analysed in the sagittal plane using customized procedures (Matlab, MathWorks, USA). From these movement data three kinematic time series were used: 1) forward-backward trunk movement of both rowers (estimated as the mean position of hip and shoulder markers) with respect to the ‘boat’ movement; 2) handle positions (estimated from the three markers placed on the handles) relative to the ‘boat’ movement; and 3) ‘boat’ position estimated from the three markers placed on the ergometer system). These data were filtered using a bidirectional second order low-pass Butterworth filter with a 15 Hz cut-off frequency.

#### Relative phase

The instantaneous spatio-temporal relation between the rowers’ trunk movements was expressed by the continuous relative phase (*ϕ*, see Eq 1). First, the continuous phase angles *θ*_*i*_, with subscript *i* depicting the stroke rower (1) and bow rower (2) respectively, were determined using

$$\theta_{i}(t)={\text{tan}}^{-1}\left(\frac{v_{i}(t)}{x_{i}(t)}\right)$$ (3)

with *x*_i_ indicating horizontal trunk position and *v*_*i*_ the trunk velocity normalized by the angular frequency for each half cycle separately, following Varlet & Richardson [37] (for similar half-cycle normalization procedures, see [38, 39]). The normalization was done because in rowing the drive (cf. backward movement) and recovery (cf. forward movement) are typically not equal in duration [8, 40], hence the backward movements had a slight but systematically higher movement frequency than the forward movements (see also [13, 41]). Start and end of each half cycle were based on the instances of the minimum and maximum excursions of the signal, which were determined using a custom made peak-picking algorithm. From the thus obtained phase angles, the continuous relative phase *ϕ*(*t*) was determined according Eq 1.

Apart from the drive and recovery differing in duration, a rower spends more time around the finish than around the catch of the stroke [13, 40], which implies that in rowing the movement cycle deviates from perfect harmonicity. Therefore, we also calculated a discrete measure of relative phase that is not sensitive to such inharmonicities. As such, for both handle and trunk movements, we determined the discrete relative phase based on point-estimates of peak excursions near the catch of the stroke, which was calculated for each full cycle as:

$$\phi_{PE}=\frac{t_{2,j}-t_{1,j}}{t_{1,j+1}-t_{1,j}}360{}^{\circ}$$ (4)

in which *t*_1,*j*_ and *t*_2,*j*_ indicate the time of the *j* ^*th*^ peak of the trunk- and handle position of rower 1 and 2.

#### Dependent measures

The data were analysed over steady state bins for each stroke rate. These bins were selected based on inspection of stroke rate time series. Evidently, each 2 *spm* change in stroke rate involved a transient stage, which in some cases lasted longer than in other cases. Hence, the bins could only be determined a posteriori. Due to the variation in transient stages, selected bins ranged between 6 and 12 strokes/bin. For each bin, the following measures were determined. Means and standard deviations of relative phase measures were determined using directional (i.e., circular) statistics [42]. As a measure of coordinative variability, we calculated the standard deviation of the discrete relative phase based on trunk positions (*SDϕ*_*PE*_). We used relative phase base on trunk position data, because displacement of the body is directly related to the displacement of the ergometers. Next to that, coordinative variability was also determined from handle movements (*SDϕ*_*PE*−*H*_), as the handle movements serve as end-effector of the rowing movement (i.e., force is applied to the water/flywheel via the handles/oar(s)). In order to minimize boat velocity fluctuations, antiphase crew coordination not only needs to be stable, but also accurate. Therefore, the absolute deviation from the intended pattern (0° or 180°) was calculated for each time sample of the continuous relative phase *ϕ*, and then averaged in each bin to obtain the absolute error of relative phase (*AEϕ*). To provide an indication of the above-described inharmonicity within the rowing cycle, for each individual rower, for each stroke rate bin, the mean ratio of the backward to forward trunk movement duration (*ratio*) was calculated. Finally, to provide insight in the effect of in- and antiphase crew coordination on ‘boat’ movements, variability of the ergometer velocity (*SDv*_*E*_) was calculated for each bin.

### Statistical analysis

Each of the above mentioned dependent measures was subjected to a 2 Pattern × 4 Tempo repeated-measures ANOVA. For this analysis we selected the first four levels of stroke rate (i.e., around 30-32-34-36 *spm*) because, as we will see, dyads achieved different maximal stroke rates in each condition, which would preclude proper comparison of all stroke rates. Furthermore, because three dyads did not achieve at least four stroke rate levels in each condition, these could not be included in this analysis. An α of .05 was adopted for all tests of significance.

## Results

All dyads but one (see below) easily managed rowing in antiphase coordination within the five-minute warm-up. In the ramp trials, a first important observation was that dyads achieved higher stroke rate levels in in-phase compared to antiphase rowing (Table 1). Still, each dyad was able to maintain antiphase at stroke rates that are similar to rates achieved in competition.

**Table 1:** Performed mean stroke rate at final bin for each dyad in in-phase and antiphase crew coordination. Dyads 4 and 8 showed a transition from antiphase to in-phase.

	Dyad nr										
	1	2	3	4	5	6	7	8	9	10	11
In-phase	39.3	37.8	42.2	35.9	46.2	48.3	41.5	39.7	39.9	44.6	44.0
Antiphase	37.6	36.8	34.6	31.3	37.0	40.9	35.9	32.9	35.8	36.7	41.9

### Coordination breakdowns

**Fig 2:**
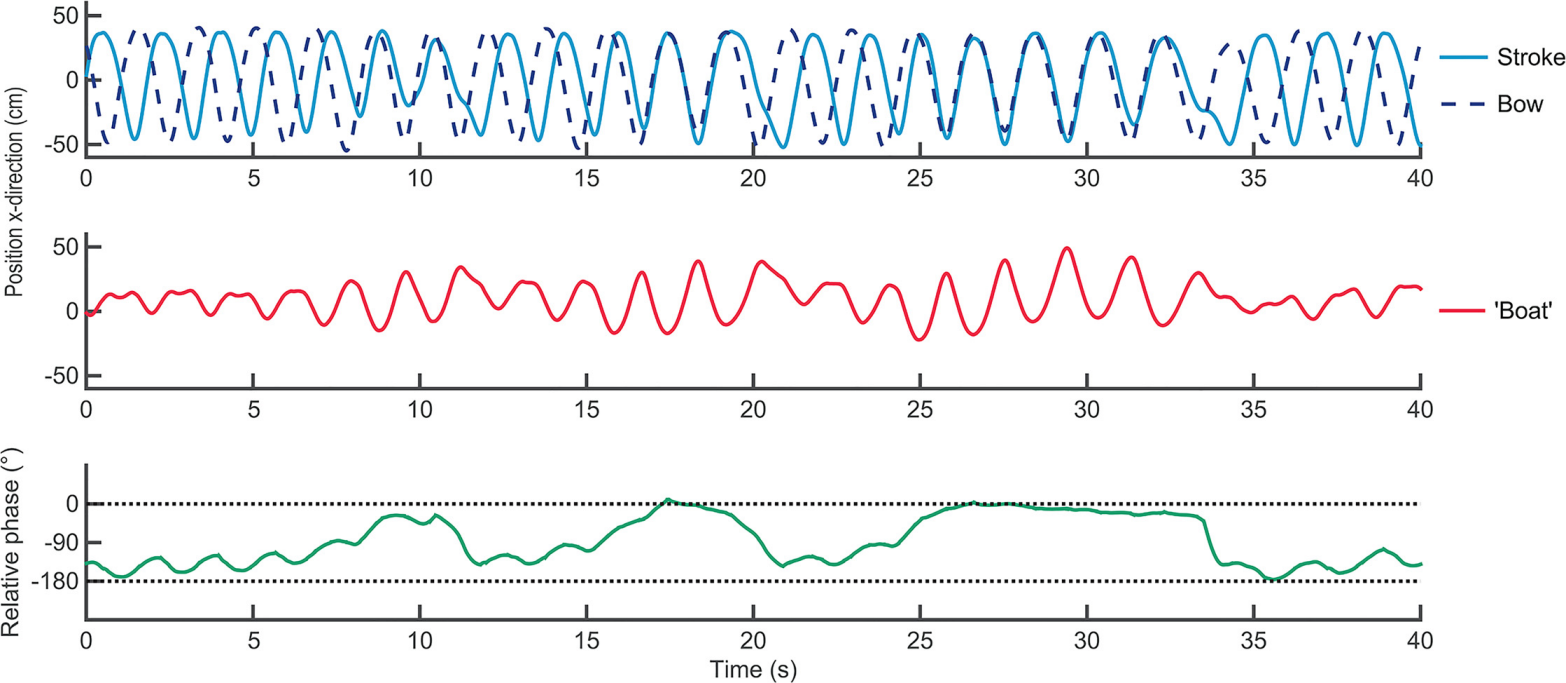
An example of a transition from anti- to in-phase crew coordination around 32 *spm*. Movements of rowers (upper panel) and ‘boat’ (middle panel), and continuous relative phase (lower panel).

Two of the eleven dyads showed a breakdown of the antiphase coordination (for an example, see Fig 2). No transitions from in- to antiphase crew coordination occurred. Notably, both cases of antiphase breakdown already occurred at the very beginning of the trial at a stroke rate between 30–33 *spm*. After the breakdown occurred, one dyad tried to restore the antiphase coordination pattern but they did not succeed, as illustrated in Fig 2. The other dyad did not make an attempt to restore coordination and they expressed verbally that they did not feel comfortable rowing in antiphase. This dyad had already shown difficulties maintaining antiphase coordination during the steady state trial and warm-up, suggesting that the coordination breakdown occurred because they were not able (or willing) to row in antiphase at any stroke rate, rather than as a result of an increase in stroke rate.

### Coordinative performance

**Fig 3:**
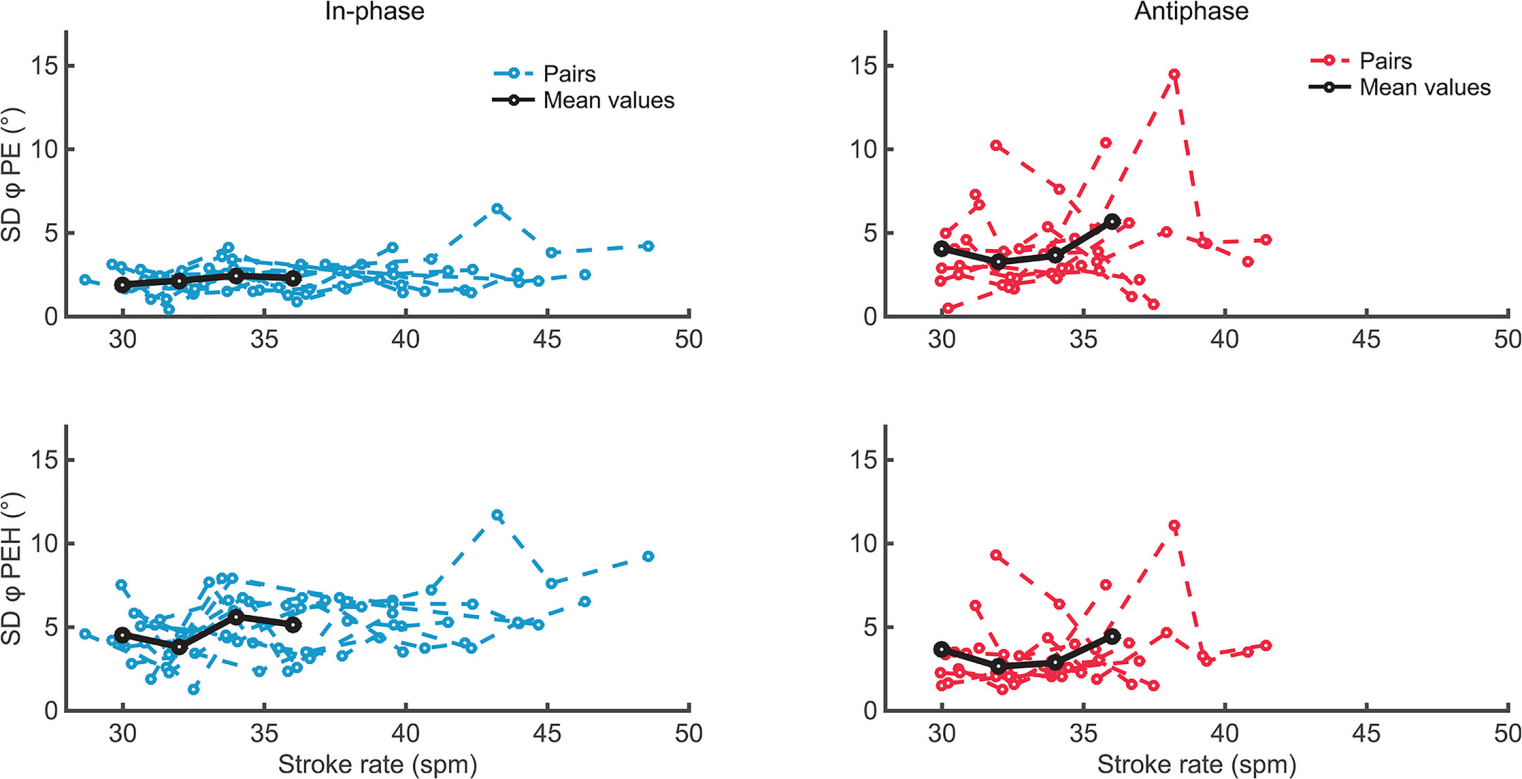
Variability of discrete relative phase. Values based on trunk movements (upper panels) and handle movements (lower panels) for in-phase (left panels) and antiphase (right panels) crew coordination depicted against performed stroke rates for all eleven dyads. Black lines indicate the means over the eight dyads as used in the RM ANOVA for stroke rates 30–36.

**Fig 4:**
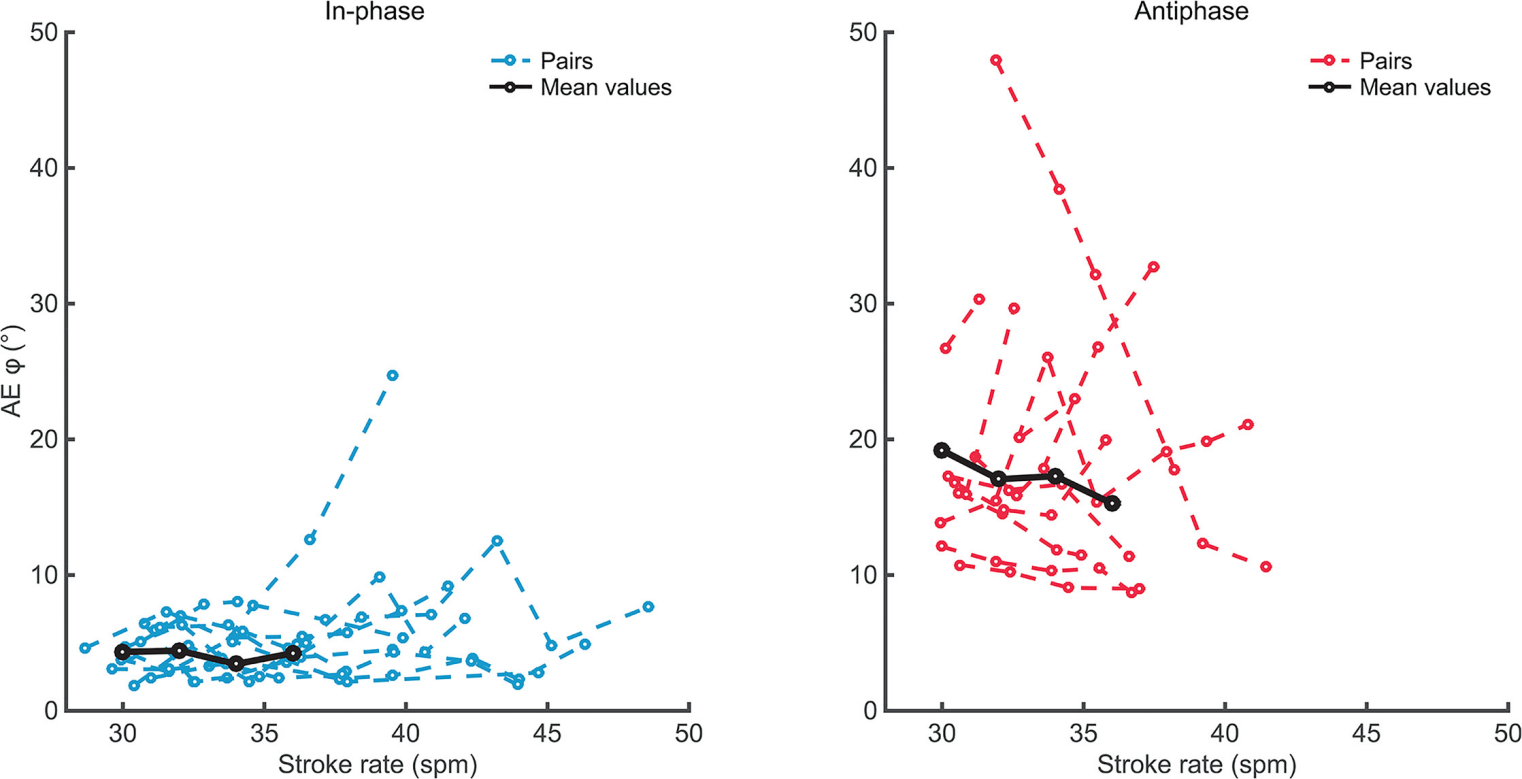
Accuracy of continuous relative phase. Absolute error of in-phase (left panel) and antiphase (right panel) crew coordination. Values depicted against performed stroke rates for all eleven dyads. Black lines indicate the means over the eight dyads as used in the RM ANOVA for stroke rates 30–36.

The effect of stroke rate on steady state crew coordination is depicted in Figs 3 and 4. For *SDϕ*_*PE*_ and *AEϕ* no significant main effect of Tempo, nor a significant Pattern × Tempo interaction was present. For *SDϕ*_*PE*−*H*_ (i.e., coordination based on the handles), a significant main effect of Tempo was found, *F*(3,21) = 3.102, *p* < .05, *η*_*p*_^2^ = .307. Post hoc analysis (Bonferroni-corrected *t*-tests) of this main effect revealed no significant differences between the four levels of stroke rate. Looking at the mean values of *SDϕ*_*PE*−*H*_ for each tempo condition (30 *spm*: 4.13°, 32 *spm*: 3.21°, 34 *spm*: 4.24°, 36 *spm*: 4.81°) the main effect suggests a slight increase in variability towards the higher stroke rate (see also Fig 3, lower panels).

Regarding the difference between in- and antiphase, *AEϕ* was higher for antiphase than for in-phase crew coordination (Table 2, see also Figs 3 and 4), which was confirmed by a significant main effect for Pattern (see Table 2). Quite remarkably, the coordinative variability (both *SDϕ*_*PE*_ and *SDϕ*_*PE*−*H*_) did *not* differ significantly between the in- and antiphase pattern (Table 2, see also Fig 3 lower panels) as there was neither a significant effect of Pattern nor a Pattern × Tempo interaction.

**Table 2:** Mean values for in-phase and antiphase rowing in terms of interpersonal coordination (*SDϕ*_*PE*_, *SDϕ*_*PE*−*H*_, and *AEϕ*), ergometer velocity fluctuations (*SDv*_*E*_) and drive-recovery ratio (*ratio*), and RM ANOVA statistics.

	Mean values		RM ANOVA results			
	In-phase	Antiphase	*F*	*df*, *error*	*p*	*η*_*p*_^2^
*SDϕ*_*PE*_ (°)	2.235	4.183	4.753	1, 7	.066	.404
*SDϕ*_*PE*−*H*_ (°)	4.813	3.438	2.366	1, 7	.168	.253
*AEϕ* (°)	4.125	17.189	21.919	1, 7	< .005	.758
*SDv*_*E*_ (m/s^2^)	0.667	0.221	931.383	1, 7	< .001	.993
*ratio*	.814	.884	44.861	1, 15	< .001	.749

### Backward-forward movement ratio

**Fig 5:**
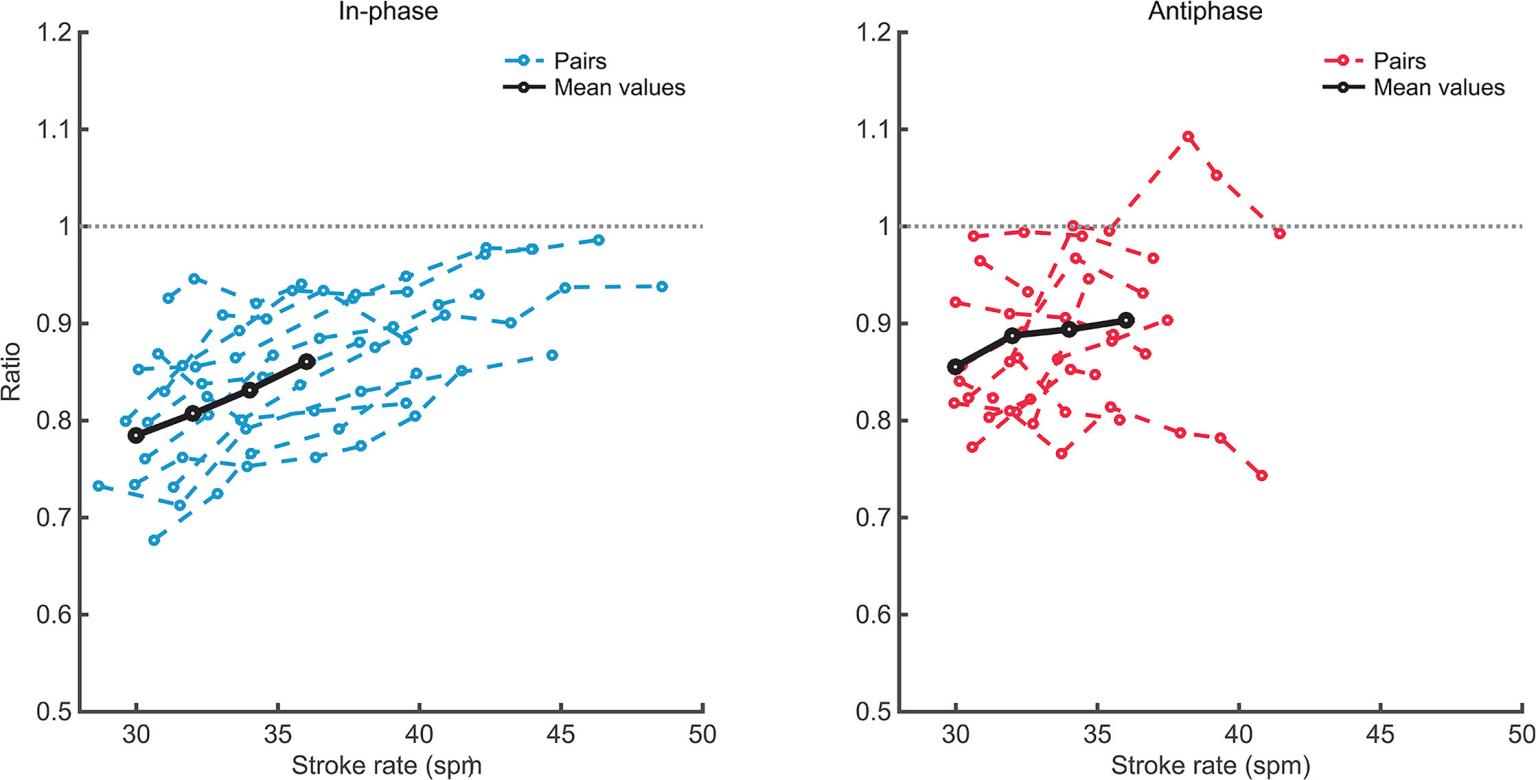
Drive-recovery ratio. *Ratio* for individual rowers in in-phase (left panel) and antiphase crew coordination (right panel), depicted against performed stroke rates for all eleven dyads. Note that for clarity of presentation, we only plotted the ratios of each stroke rower (i.e. rower 1). Black lines indicate the means over the sixteen rowers as used in the RM ANOVA for stroke rates 30–36.

Similar to De Brouwer et al. [13], trunk movements of the rowers were slightly faster during the drive than in the recover, for both in- and antiphase crew coordination. This was reflected by the *ratio* values that were generally lower than 1. From Fig 5A it seems that the *ratio* increased with stroke rate, especially for in-phase rowing, indicating that the rowing cycle became more harmonic. This was supported by the results from a 2 Pattern × 4 Tempo repeated-measures ANOVA over the first four tempo levels (see *‘Statistical analysis’*) for the *ratio* of both rowers of these 8 dyads (n = 16). This analysis revealed that the *ratio* was significantly higher (i.e. closer to 1) for the antiphase pattern (see Table 2) and that for both in-phase and antiphase crew coordination the *ratio* increased with tempo for 30–36 *spm*, given the main effect of Tempo *F*(1.50,22.55) = 19.89, *p* < .001, *η*_*p*_^2^ = .570. The Pattern x Tempo interaction did not reach significance.

### Movements of the ergometer system (‘boat’)

**Fig 6:**
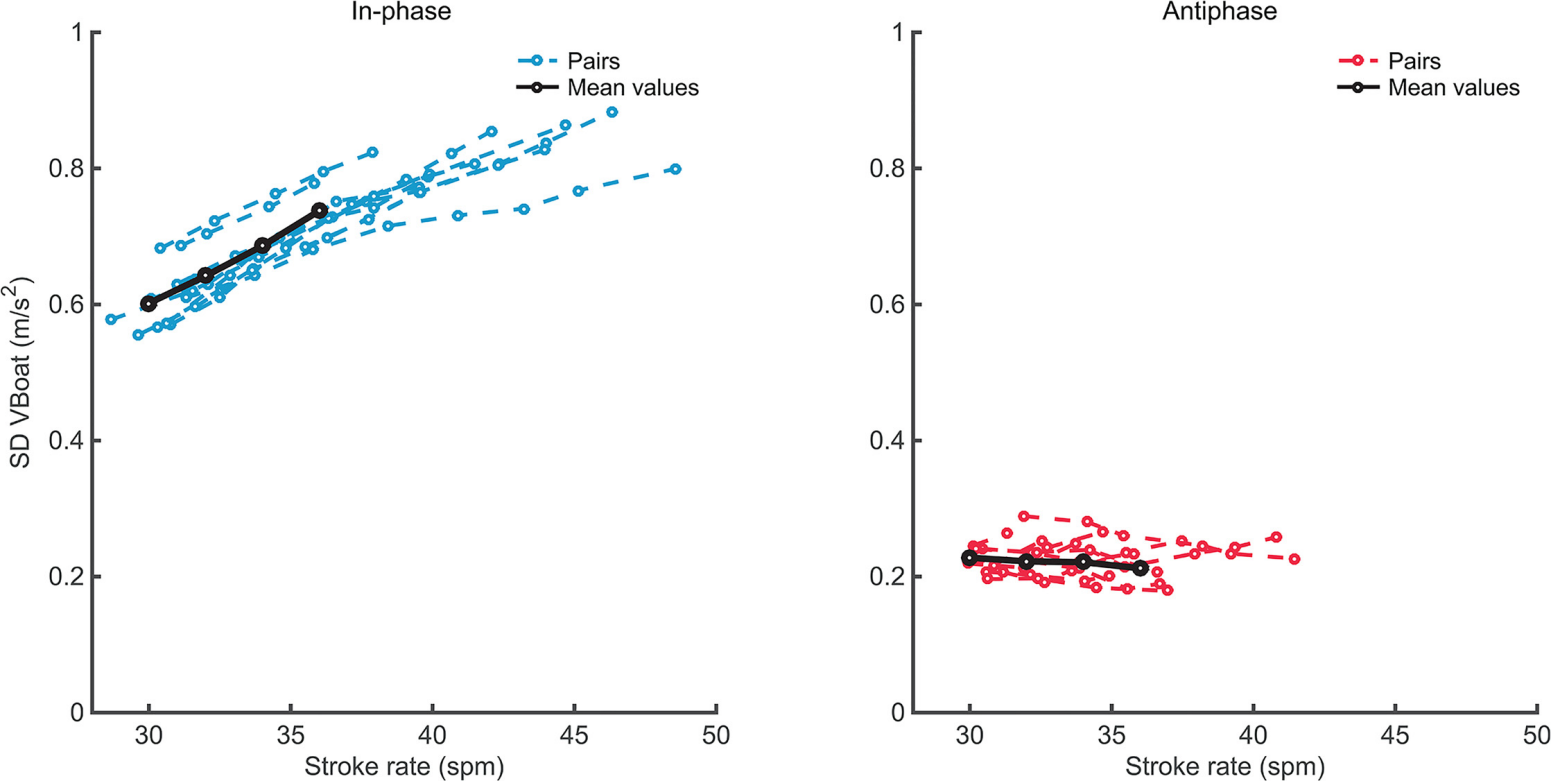
Variability of ‘boat’ velocity. Values for in-phase (left panel) and antiphase (right panel) crew coordination depicted against performed stroke rates for all eleven dyads. Black lines indicate the means over the eight dyads as used in the RM ANOVA for stroke rates 30–36.

**Fig 7:**
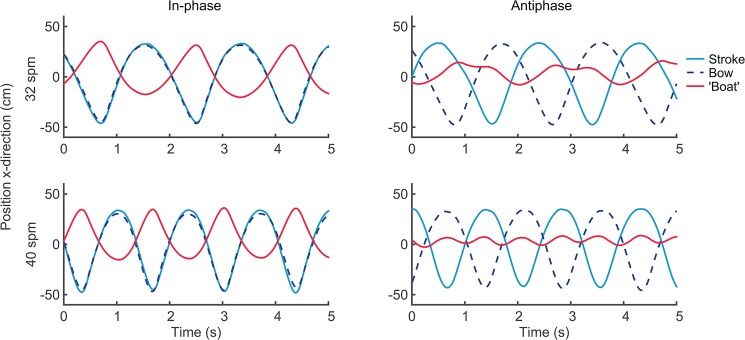
Example of movements of rowers and ‘boat’. Example of in-phase (left panels) and antiphase (right panels) crew coordination at 32 *spm* (upper panels) and 40 *spm* (lower panels).

Fig 6 shows that the fluctuations in ergometer velocity, as indicated by *SDv*_*E*_, were much lower for antiphase than for in-phase crew coordination, yielding a significant main effect of Pattern (see Table 2). Furthermore, whereas for in-phase *SDv*_*E*_ clearly increased with stroke rate, Fig 6 suggests that for antiphase crew coordination this was not the case. This was corroborated by a significant Pattern × Tempo interaction, *F*(3,21) = 74.93, *p* < .001, *η*_*p*_^2^ = .915. Analysis of the simple effects confirmed a significant effect of Tempo for in-phase, *F*(2.04,11.36) = 152.38, *p* < .001, *η*_*p*_^2^ = .956, but not for antiphase, *F*(1.75,12.24) = 1.59, *p* = .243, *η*_*p*_^2^ = .185. These effects of pattern and tempo on ergometer movement can also be observed in Fig 7, which provides examples of the rowers’ trunk movements and the ergometer movement during a few strokes of in-phase and antiphase rowing at 32 *spm* and at 40 *spm*.

## Discussion

This study examined in- and antiphase crew coordination at increasing stroke rates on mechanically linked ergometers. The main aim was to investigate whether with an increase in stroke rate antiphase crew coordination could still be maintained stably or that it would break down into the more stable in-phase pattern, as may be expected from coupled oscillator dynamics. Antiphase breakdowns were observed for two of the eleven dyads, whereas no coordinative breakdowns were seen for in-phase crew coordination. In antiphase, dyads did not achieve stroke rates as high as in in-phase crew coordination. Still, the study shows that most dyads were able to sufficiently maintain antiphase crew coordination on linked ergometers at stroke rates as high as used in on-water racing.

### Coordination breakdowns

Two dyads showed a breakdown from anti- towards in-phase crew coordination. These transitions already took place in the very beginning of the ramp trial at 30–33 *spm*, which might suggest that the breakdowns were not necessarily induced by an increase in movement rate. In line with this reasoning, one of these dyads expressed trouble with antiphase crew coordination in general (see ‘*Results’*), while the other dyad tried to restore the antiphase pattern, but failed to do so. As can be observed in Fig 2, once antiphase crew coordination is lost, the ‘boat’ starts moving at larger amplitudes and it is difficult to counter these boat movements to regain antiphase (see below for more detailed discussion: ‘*On mechanical coupling‘*). Interestingly, after the experiment the latter dyad mentioned that their initial antiphase breakdown (starting around the 6^th^ s of the trial, see Fig 2) might have occurred because they temporary lost their attention/concentration. This suggestion fits with earlier findings that antiphase coordination requires more attention than in-phase coordination, also in interpersonal tasks [43]. It could well be that the observed breakdowns were instigated by perturbing sources that were not related to an increase in tempo. According to coupled oscillator dynamics, at higher movement frequencies the coordination is less resistant to (either external or internal) perturbations, especially for antiphase coordination [44]. As in the present experiment the ramp trials already started at a reasonably high stroke rate (30 *spm*), at this tempo, the coordination might already have been too sensitive to (e.g., attentional) perturbations for these two dyads.

In contrast to the two dyads that showed breakdowns of their antiphase rowing patterns, the remaining nine dyads were able to achieve antiphase crew coordination at rates maxing out at 34 up to 42 *spm* (see Table 1). In antiphase crew coordination these nine dyads did not achieve stroke rates as high as in in-phase rowing. In principle, this might indicate that if they would have been able to reach higher rates, antiphase breakdowns might have occurred. It is worth mentioning that in previous experiments on transitions from anti- to in-phase coordination, participants often were instructed ‘not to resist if they felt that the pattern tended to change’ (e.g., [16, 18]). In the present experiment we simply instructed dyads to perform the instructed patterns up to the fastest as possible rate. In other words, dyads may have decided to stop when they felt they could not maintain the antiphase pattern anymore at a certain tempo.

### Achieved stroke rates

An additional explanation for the higher stroke rates attained in in-phase as compared to antiphase crew coordination might be found in a difference in ‘boat’ movement in these two situations. The difference between the maximum tempos in both patterns may (at least) partly be due to the ergometer setup. As the slides allow the ergometers to float free with respect to the floor, in in-phase the crew concurrently moves the ‘boat’ backward and forward because the weight of the ‘boat’ is much lower than that of the crew (see also *Introduction*and Figs 6 and 7). Previous research indicated that moving the light ergometer rather than the heavy body makes it easier to achieve higher stroke rates compared to rowing on an ergometer that is fixed to the floor [45]. For antiphase coordination, however, the forces and, hence, movements of the rowers counteract each other and the system of ergometers stays approximately at the same place (Figs 6 and 7). Thus, during antiphase rowing the free-floating ergometers behave as fixed ergometers, which can partly explain why achieved stroke rates were lower for antiphase rowing.

In the study of De Brouwer et al. [13] a servomotor was used to resist the movement of the ergometers, that is, to induce the velocity dependent resistance that is present on the water. In the current experimental set up, however, we did not use such an ‘extra’ resistance. Therefore, for in-phase rowing the ‘boat’ could be moved with more ease compared to De Brouwer et al. [13] and compared to on-water rowing, which indicates that the highest tempos reached in in-phase in the present experiment likely overestimate on-water stroke rates. For antiphase rowing this was less of an issue because the ‘boat’ stayed approximately still. Together, the higher stroke rates achieved in in-phase rowing are at least in part due to 1) increased movements of the ergometer system, and 2) the fact that there was no resistance applied to the moving ergometers.

### Steady state coordinative performance

As mentioned before, we expected that for both patterns the crew coordination would deteriorate with increasing stroke rate, with a stronger effect for antiphase than for in-phase. Although we found a significant main effect of stroke rate on the consistency of the coordination between handle movements (as indexed by *SDϕ*_*PE*−*H*_), the effect was rather small. If any, crew coordination variability slightly increased towards the higher frequencies (see Fig 3). This interpretation would be in line with expectations based on the coupled oscillator model (see ‘Introduction‘). That said, interpersonal pendulum swinging experiments demonstrated that at high movement rates coordinative variability indeed increased with tempo, while at very low movement rates (i.e. rates far below the eigenfrequency of the pendulum) coordination *also* deteriorated [19]. This may be taken to suggest the existence of a stroke rate at which crew coordination is optimal and that at lower stroke rates crew coordination might also become less consistent. Note that in the present study we compared ergometer rowing at rather high stroke rates (namely > 30 *spm*). Regarding on-water rowing, there are indeed some indications that (in-phase) crew synchronization is poorer for stroke rates < 30 *spm*. A study by Hill [8] reported that the mean synchronization offset of the catch was 14.2 *ms* for endurance rowing (23–25 *spm*) and 11.2 *ms* for intensive rowing (31–41 *spm*). Thus, for lower stroke rates, it remains to be examined whether there is a negative (as predicted by the coupled oscillator model [17]) or positive relation between stroke rate and coordinative performance. In sum, however, there was no compelling support that steady-state coordinative performance (for both in- and antiphase) substantially changed over different stroke rates, given that the tempo effect on handle coordination was rather marginal and also given the lack of significant tempo effects in *SDϕ*_*PE*_ and *AEϕ*.

For most dyads antiphase crew coordination seemed sufficiently stable to maintain at the highest possible movement rates, which is a promising message for potential on-water application. In line with previous findings [13], in-phase crew coordination was more accurate than antiphase crew coordination. Notably, for rates around 36 *spm* (i.e., the rate used in [13]) both the variability and accuracy values (see Figs 3 and 4) were reasonably smaller than in De Brouwer et al. [13], especially for antiphase rowing. A surprising finding was that coordinative variability (as indexed by *SDϕ*_*PE*_ and *SDϕ*_*PE*−*H*_) was *not* significantly higher for antiphase coordination, which is counter to previous findings in rowing [13] and ample evidence from interpersonal coordination dynamics [4, 18–20]. This finding indicates that the dyads in the present off-water study coordinated the catch in in- and antiphase rowing with comparable consistency. On water, the oars serve as the end-effectors of the rowing movement, as forces are transferred to the water via the blades. Similar to this, on the ergometer, forces are transferred to the flywheel through the handle, which motivated us to analyse the coordination between the handle movements as well. Note, however, that oar(s) are much heavier and involve more inertia than the ergometer handles. As such, it is uncertain whether on-water oar coordination would yield similar results.

In the present study, rowers adapted their individual rowing strokes to the antiphase pattern, given that their backward-forward movement ratios suggested more harmonic rowing cycles in the antiphase than in the in-phase condition. Notably, De Brouwer et al. [13] found no difference between in- and antiphase crew coordination in this respect. The backward trunk movement (drive phase) was faster than the forward trunk movement (recovery phase), as reflected by the *ratio* values that were generally lower than 1 (Fig 5). Because for antiphase crew coordination the drive of one rower coincides with the recovery of the other rower, the inherent backward-forward asymmetry (i.e., *ratio* < 1) in the cycle of each of the two individual components introduces extra fluctuations in their continuous relative phase (see Fig 7), which is captured in the lower accuracy of coordination in antiphase (as indicated by *AEϕ*, see Table 2 and Fig 4). Hypothetically, if the movements would be perfectly harmonic (although given the nature of the rowing stroke this is practically impossible) all boat velocity fluctuations may cancel out. In this context it is noteworthy that, on average, in antiphase the movements were indeed more harmonic (e.g. indicated by *ratio;* see Fig 5) than for in-phase crew coordination, indicating that the rowers adapted their rowing strokes in favour of the crew’s coordination pattern.

For both in- and antiphase crew coordination the trunk movement became more harmonic (indicated by *ratio*) with an increase in stroke rate. For in-phase crew coordination this is in line with indications from on-water rowing which showed that at increasing stroke rates the recovery phase shortens relative to the drive phase (e.g., [8]). Because the less harmonic movements at lower stroke rates introduce more boat fluctuations (especially around the catch and finish, see also [13]), this may counteract efficiency-related benefits of antiphase crew coordination [13, 15]. In fact, it can be expected that at the lower stroke rates, for instance, during endurance practice (18–24 *spm*) such benefits of antiphase rowing diminish. Because of these and other reasons (see above), it might be interesting to investigate in- and antiphase crew coordination at strokes rates < 30 *spm*.

### On mechanical coupling

Next to being perceptually coupled, the rowers in the experiment were physically connected via the ‘boat’. Mechanical coupling differs from perceptual coupling (e.g., haptic, auditory and visual coupling) in that it is not possible to escape from: the body of each agent gets passively shaken by the movement of the other agent [28], whereas perceptual coupling is mediated by the degree to which an agent is sensitive to, or able to detect the pertinent information [46], for instance by means of attention devoted to the information source [4, 46]. As an extreme example of the latter, one could simply close the eyes to escape from visual coupling [32]. This implies that the mechanical coupling might be more stringent than perceptual coupling. Although many natural tasks involve mechanically coupled coordination, for instance shaking hands, dancing the tango, or jointly moving furniture, (cyclic) coordination of mechanically coupled humans has been relatively unexplored in scientific literature (however, see, e.g., [33, 47]). Note also that a direct physical link between humans always implies kinaesthetic (i.e., haptic and proprioceptive) coupling, but that kinaesthetic coupling can also be present without mechanical coupling. When dyads coordinate their actions on the basis of haptic information see also [48], it has been shown that they amplify their forces to generate a haptic information channel [49, 50]. A similar principle may hold true for crew rowing, as Hill [8] suggested that an increase in force output in crew rowing provides a better kinaesthetic perception via the boat, which may facilitate the mutual adaption of force patterns.

As noted, when antiphase coordination is lost it is challenging to return to this pattern, because once the ‘boat’ starts oscillating at larger amplitudes it is difficult to counter these movements (Fig 2). In fact, we might consider this mechanical link to share similarities with the wall vibrations that underlay the synchronisation of two pendulum clocks as Christiaan Huygens observed in 1665. As a result of the exchange of energy through mechanical vibrations via the wall, the initially uncoordinated clocks became coordinated over time in either in-phase or antiphase coordination [51]. Relatedly, in experiments with metronomes placed on a freely moving base, transitions from antiphase to in-phase occurred (e.g., [52]—many movies are available on-line in which synchronizing of metronomes is demonstrated, of which arguably the most illustrative can be found here www.youtube.com/watch?v=yysnkY4WHyM and here www.youtube.com/watch?v=kqFc4wriBvE). In this non-living system, this is due to the mechanical coupling via a third part that can move, namely the freely moving base. It is important to recognize that while in perfect antiphase the metronomes’ movements completely cancel out the movement of the base, this does *not* mean that there is no coupling, or less coupling than in in-phase. In fact, in antiphase the *effect* of the coupling is smaller, in that the base does not move, but once it starts moving (due to some internal or external perturbation) the base movements influence the metronomes’ movements, causing them to reside into in-phase synchronization with the base and, thus, with each other. Hence, a similar mechanical interpersonal link may form a strong source for attraction to in-phase that is arguably more stringent than for perceptual coupling (cf. [28]). In line with this, our study demonstrated that transitions in between-person coordination occur not only in purely visually coupled humans [18], but also when they are mechanically linked. Similar to the metronomes, in the present experiment the movements of each rower (cf. metronome) set the ‘boat’ (cf. base) in motion (see Fig 2), thereby mechanically influencing the other crew member. In that sense, both in intra- and interpersonal coordination, mechanical coupling may be considered as a source of perturbation that requires anticipatory movements [53]. Alternatively, it may also stabilise coordination by constraining the movements of the coupled agents [33].

### Movements of the ergometer system (‘boat’)

The effect of crew coordination pattern on velocity fluctuations of the ‘boat’ was evident (see e.g. Fig 7). Antiphase crew coordination resulted in a reduction of about 60% in velocity fluctuations of the ‘boat’, consistent with earlier findings [13]. Moreover, for in-phase rowing an increase in stroke rate resulted in an increase in velocity fluctuations of the ‘boat’ (cf. [12]), while this was not the case for antiphase rowing. These results suggest that the effect of antiphase crew coordination on boat velocity fluctuations becomes even more beneficial at higher stroke rates.

### Generalisation to on-water rowing

Although ergometer rowing is not exactly the same as on-water rowing, the results of the present study do allow to be generalised to on-water rowing. Nevertheless, it is important to take into account that, for instance, there are no lateral and vertical (angular) movements of the boat, handles are used rather than oars (which have a certain length and weight), and oar handling technique and hydrodynamics around the blades are not present in an ergometer task. Furthermore, regarding the coupling underlying the synchronisation of the crew, in interviews rowers have reported that they use several information sources to coordinate their movements, such as the looming of the rower in front of them, the haptic channel and physical connection between rowers via the boat, and the flow of water running past the boat [54]. Evidently, the latter was not present in the ergometer setup. Nonetheless, although these issues were beyond the scope of the present study, testing crew coordination on-water is the next important step.

## Conclusion

Although the experiment was initially set up to invoke breakdowns of antiphase coordination, only two transitions from anti- to in-phase occurred in at the very beginning of the ramp trial, whereas the other dyads easily maintained the antiphase pattern at increasing stroke rates. A striking finding was that antiphase crew coordination was not significantly more variable than in-phase crew coordination. Together, the results suggested that although antiphase crew coordination is less stable (i.e. less resistant to perturbations) than in-phase crew coordination, it is certainly possible to maintain antiphase rowing at a sufficiently consistent and accurate level at high movement rates. In fact, we only found minor indications that crew coordination may deteriorate at increasing tempos. In addition, antiphase rowing implies a significant reduce in velocity fluctuations of the ‘boat’, an effect that grows even stronger with increased stroke rate. This might suggest that benefits of antiphase rowing may actually *in*crease with stroke rate.

## Supporting Information

S1 Dataset Data of analysed variables of all dyads. (XLSX)

S1 Movie Exemplary movie of a dyad rowing in-phase at 30 and 36 *spm*. (MOV)

S2 Movie Exemplary movie of a dyad rowing antiphase at 30 and 36 *spm*. (MOV)
